# Head and neck cancers in France: an analysis of the hospital medical information system (PMSI) database

**DOI:** 10.1186/1758-3284-2-22

**Published:** 2010-09-01

**Authors:** Jean Lacau St Guily, Isabelle Borget, Alexandre Vainchtock, Vanessa Rémy, Claire Takizawa

**Affiliations:** 1 Service ORL et Chirurgie Cervico-Faciale, Hôpital Tenon, UFR et Université Pierre-et-Marie Curie-Paris 6, Paris, France; 2 Biostatistic and Epidemiology Department, Institut Gustave Roussy, Villejuif, France; 3 HEVA, Lyon, France; 4 Sanofi Pasteur MSD, Lyon, France

## Abstract

**Background:**

With 16,005 new cases and 5,406 related deaths in 2005, France is particularly concerned by Head and Neck (H&N) cancers. In addition to tobacco and alcohol, Human Papillomavirus (HPV) has been reported as a risk factor for H&N cancers. The literature on the burden of these cancers in Europe is scarce. This study was performed to assess the medical and economical burden of hospitalisations for H&N cancers in France.

**Methods:**

The French national hospital database (PMSI), in which admissions to public and private hospitals are recorded, was retrospectively analysed to assess the annual number of patients hospitalised for H&N cancers and associated hospital costs from the healthcare payer perspective. ICD-10 codes (16 codes classified as oral cavity, oropharynx, pharynx, salivary glands and larynx) were used to extract admissions for these cancers. Hospital stays, chemotherapy and radiotherapy sessions were extracted to assess patients' management. Costs of admissions were obtained from French official tariffs.

**Results:**

In 2007, there were 36 268 patients hospitalised for H&N cancers, of whom 81% were men, corresponding to 60 200 hospital stays and 287 846 sessions of chemo- or radio-therapy. Oropharynx cancer was the most frequent (28% of patients), followed by oral cavity cancer (25% of patients). The peak of frequency was observed in the 55-59 years age group. Patients were mainly treated in medicine (48%) and surgery (23%) units. Mean annual cost per patient ranged from €2 764 to €7 673 leading to a total hospital cost of €323 millions in 2007 (including hospitalization and expensive drugs). With 26% of H&N cancers attributable to HPV infections, 9 430 patients were hospitalized due to HPV-related H&N cancers, representing €138 million in 2007.

**Conclusion:**

Even without taking into account the rehabilitation costs, the hospital burden of H&N cancers is considerable.

## Background

In Europe, head and neck cancer (H&N) accounted for approximately 143 000 new cancers in 2007 and were responsible of more than 68 000 deaths [1]. They have broadly varying rates of incidence and mortality around the world. In North America and Europe, tumours usually arise from the oral cavity, oropharynx, or larynx, whereas nasopharyngeal cancer is more common in the Mediterranean countries and in the Far East. France is particularly concerned by head and neck cancers [2]. With 16 005 new cases in 2005, they represented 3.8% of all incident cancer cases. They are the fourth highest incidence of cancers in men and the eleventh highest incidence in women. In terms of mortality, 5 406 related deaths occurred in 2005, including 82% in men. It represented 2.7% of annual cancer deaths and the tenth more lethal cancer [3,4].

These cancers are more common in men and in people over age 50. Head and neck cancer refers to a group of cancers originating from the upper aerodigestive tract, including oral cavity, oropharynx, pharynx other than oropharynx (nasopharynx, hypopharynx), larynx, salivary glands, and other sites located in the head and neck area. Head and neck cancers are strongly associated with environmental and lifestyle risk factors. Use of tobacco is the largest risk factor for H&N cancers [5,6]. During the past two decades, the role of tobacco in the incidence of head and neck cancer has however decreased, thanks to public health efforts at tobacco control and reduction in the prevalence of cigarette smoking. Alcohol consumption is frequently associated with tobacco use as a co-factor in oncogenic risk, especially in oral cavity and hypopharynx cancers.

Moreover, numerous studies have highlighted the increasing role of the sexually transmitted human papillomavirus (HPV) in the incidence of H&N cancers. Contribution of HPV in H&N cancers varies according to tumour site location. Most HPV positive cancers occur in the oropharynx [7], in particular tonsil and base of the tongue. HPV could account for 33% to 72% of oropharynx cancers [8,9]. Tonsillar cancers have been reported to have the highest prevalence rate of HPV-DNA (more than 50%) [10-12]. HPV positive rates are lower in other localizations, ranging from 5 to 16% for larynx [13,14], 24% in a report including hypopharynx carcinomas in larynx [8,15] and 23.5% in oral cavity. The wide variations in HPV prevalence which are reported may depend on the HPV diagnostic methodologies, especially in earlier studies.

Treatment of head and neck cancer depends on the initial localization of the tumor, on patient's comorbidity and on potential side effect of treatment. Surgical resection, radiotherapy, radiochemotherapy, induction chemotherapy, radiobiotherapy (with anti epidermal-growth-factor receptor (EGFR) like cetuximab and other anti EGFR), are the therapeutic methods in locally advanced cases, the most frequent mode of presentation of H&N cancers. The overall prognosis remains poor. In addition, patients frequently suffer from other co-morbidities. A number of advances in therapies for head and neck cancers have been developed in the past several years, including the availability of anti-EGFR agents, induction chemotherapy, new microsurgical and reconstructive surgery techniques or accelerated radiotherapy.

The economic impact of head and neck cancers is poorly documented. The cost of illness has been estimated for some specific countries, like the UK [16], the USA [17], the Netherlands [18], Greece or Germany [19], using various methodologies. Moreover, as these cancers presented great variation in terms of localisation and incidence rate worldwide, the use of economic estimations performed in others countries may be hazardous. In 2004, the French National Institute of Cancer (Institut National du Cancer-INCa) has estimated that the global direct medical costs related to cancer amounted to 11 milliards euros (Md€), including 7 Md€ due to hospitalisation costs, whereas indirect costs due to loss of productivity and premature death were estimated at 17 Md€ [20].

The specific cost related to head and neck cancers in France has however never been documented. The objective of the present study was to assess the annual number of patients hospitalised for head and neck cancers in France and to estimate the economic burden associated with their management.

## Methods

### Data sources

A retrospective analysis was performed using data extracted from the French Medical Information System (Programme de Médicalisation des Systèmes d'Information - PMSI) for 2007 which covers all French public and private hospitals, except military and psychiatric hospitals. In fact, since 2004, all French hospitals has adopted a prospective payment system based on case-mix, called "Tarification à l'Activité". Each hospital stay resulted in the production of a standard discharge summary ("Résumé Standard de Sortie" RSS) following inpatient conventional stays, day-hospital stays or sessions. The RSS contains information on the nature of the treatment and work-up (the examinations) carried out during the stay, on the main diagnosis that led to the hospital admission, on co-morbidities or possible complications. Diagnoses are coded using the International Classification of Diseases, 10^th ^revision (ICD-10) either as primary, related, or significant associated diagnosis. The RSS is then integrated into a Diagnosis Related Group (DRG) used for classification of hospital stays.

All the hospital stays and sessions performed during a specific year are summarized into standardized discharge reports and collected in a national database called PMSI. This database is the basis of hospitals funding, but also allows to estimate various indicators by disease, like the number of patients treated annually, the number of stays per patient or the burden of the disease, with an exhaustiveness close to 98% in 2007 [21].

The SAE database (''Statistique Annuelle des Etablissements de santé'') [22] relies on information collected through an exhaustive and compulsory survey covering all French hospitals. It was used to extract data on radiotherapy sessions for private sector, as they are not available in the PMSI database.

Since the introduction of a DRG type prospective per case payment in 2004, a list of so-called expansive drugs has been set. Conversely to other drugs whose cost are included in the DRG tariff, drugs on the list are reimbursed 100% to the hospital based on a national reference reimbursement tariff and the EMI, conditional to the adherence of prescription recommendations. The FICHCOMP database contains the expenses of drugs on the list, by drug and per stay (for public hospitals only).

### Data collection

Cases were extracted from the PMSI database using the ICD-10 codes referring to head and neck cancers. They were classified into five categories corresponding to five localizations: oral cavity (including lip, tongue, gum, floor of the mouth, palate, and mouth; coded as C00, C02-06), salivary glands (including parotidis glands, salivary glands; coded as C07-08), oropharynx (including base of tongue tonsil, oropharynx; coded as C01, C09-10), pharynx other than oropharynx (including rhinopharynx, pyriform sinus, hypopharynx, others; coded C11-14) and larynx (coded as C32).

### Number of stays

The annual number of stays was assessed after extraction of all hospital stays with one of the above-mentioned head and neck cancers as a primary diagnosis in the hospital database. Since a single hospital stay may include other ICD codes with head and neck cancers coded as related or significant associated diagnosis (and not primary diagnosis), a medical interpretation by a PMSI specialist was required to assess whether the specific hospital stay had a direct link with the diagnoses of interest. All medical stays were classified according to the type of management: medical, surgical, exploration or palliative care. Distinction was made between full stays and one-day hospital setting (defined as hospitalizations of less than 48 hours). Diagnosis Related Groups (DRG) were evaluated, as well as the length of stay for full stays.

### Number of chemotherapy and radiotherapy sessions

The number of sessions referred both to chemotherapy and radiotherapy sessions. Data on chemotherapy sessions were extracted from the PMSI database, by selecting all hospital stays with a primary diagnosis coded as Z51.1 (''Chemotherapy session of neoplasm'') and an associated or a related diagnosis coded as a head and neck cancer. In the same way, data on radiotherapy sessions were extracted using the Z51.0 code (''Radiotherapy session'') as primary diagnosis. It only concerned the radiotherapy sessions performed in the public sector (radiotherapy sessions performed in the private sector are usually not reported in the PMSI database). The SAE database was used to estimate the overall annual number of radiotherapy sessions performed in public and private hospitals in 2007 and to calculate the ratio between public and private radiotherapy sessions. The annual number of radiotherapy sessions performed in the private sector was then estimated by applying the ratio to the number of radiotherapy sessions for head and neck cancers retrieved in the PMSI database in the public sector.

### Number of patients

Since a patient may have several hospital stays or sessions during a year, the number of patients hospitalized at least once in 2007 for head and neck cancers was obtained by linking all hospital stays and sessions, based on patient's identification number. This number is built using the patient's social security number, date of birth and gender. After anonymisation, data are sent to the regional agency of hospitalization ("Agence Régionale d'Hospitalisation - ARH"). This number allows linking all hospital stays that occurred in public and private sectors in 2007 by patient (except for radiotherapy session performed in the private sector). It is then possible to estimate the total number of head and neck cancer patients that were hospitalized at least once in 2007, by sex and age group. In 2007, respectively 98% and 87% of all hospital stays and sessions were successfully chained, meaning that the total number of patients with radiotherapy or chemotherapy sessions was slightly underestimated.

### Economic evaluation

Costs were considered from the healthcare payer perspective. It included hospitalization costs and expenses of innovative drugs, related to public and private sectors. Ambulatory costs and indirect costs related to productivity loss were not considered in the main analysis.

Hospital costs were calculated using the official 2008 diagnosis related group (DRG) tariffs for public hospitals (that were the tariffs available when the analysis was performed). Tariffs included nursing care, treatments, drugs, accommodation and investment costs for hospitalized patients. For public hospitals, it also covers medical and technical acts. For private hospitals, costs were estimated using the official 2008 DRG tariffs for private hospitals to which physician's fees were added as they are not included in private DRG tariffs and are reimbursed on a fee-for-service basis (source: ENCC 2006)[23]. Since no data are available on the cost for radiotherapy session in the private setting, its cost was estimated using those calculated for the public sector. Costs are presented as mean annual cost per patient (based on patients for whom data were firmly linked) and total cost per year (for all patients hospitalized in 2007) and were expressed by gender, cancer localization, type of care and sector (public or private).

The economic impact of expensive drugs was estimated by linking the chemotherapy sessions or stays for palliative care extracted from the PMSI database with the FICHCOMP database. It allows evaluating the proportion of these stays including the administration of an expensive drug, as well as the cost of expensive drugs. Since the FICHCOMP database was accessible for the first time for the year 2008, this was performed by using H&N cancers related stays of 2008 (by using the same methodology than in 2007).

The economic burden of H&N cancers attributable to HPV infection was estimated by using the specific prevalence of HPV infection by localization (data extracted from the international published literature, no available data for salivary glands and pharynx cancers other than oropharynx).

## Results

Table 1 gives the number of hospital stays and sessions and the number of patients hospitalized for head and neck cancer in France in 2007.

**Table 1:** Annual number of patients, hospital stays and sessions in 2007

	Number of stays (% of PD)	Number of sessions	Number of patients*			Men (%)
				
			Public	Private	Global	
**Oral cavity**	15,093 (70%)	57 294	7,685	3,803	10,786	75

**Salivary glands**	1,884 (73%)	11 841	1,218	704	1,831	61

**Oropharynx**	16,519 (60%)	99 618	8,558	4,813	12,232	82

**Pharynx**	14,358 (56%)	70 753	6,981	3,586	9,718	87

**Larynx**	12,346 (72%)	48 339	6,714	3,557	9,516	88
**Total**	60,200 (57%)	287 846	25,987	14,788	36,268	81

* As some patients may have been hospitalized for several cancers, the total number of patients is not equal to the sum of the number of patients by localization. The global number of patients is also not equal to the sum of patient treated in public and private sector, as some patients were treated in both sector.

PD = Primary Diagnosis

### Number of patients

**Figure 1:**
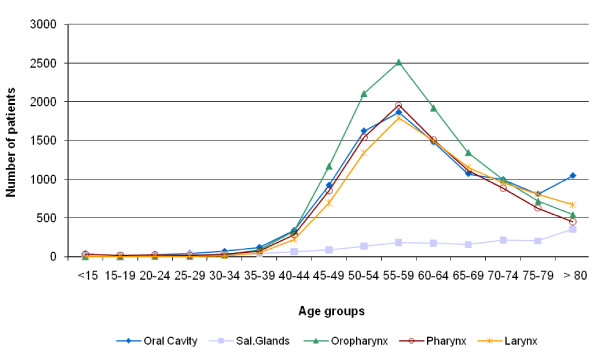
**Age distribution of patients with head and neck cancers**.

In 2007, 36 268 patients were hospitalized for head and neck cancer in France. The most frequent localization of cancer was the oropharynx (n = 12 232), followed by the oral cavity (n = 10 786), the pharynx other than oropharynx (n = 9 718), the larynx (n = 9 516) and the salivary glands (n = 1 831) (Table 1). Most of the patients were males (81%), but the proportion of men varied from 61% for salivary glands cancer to 88% for larynx cancer. For most H&N cancers, incidence increased with age until the group age of 55-59 years and then decreased. The scheme is slightly different for salivary gland cancer whose incidence is increasing with age (Figure 1).

### Number of stays and sessions

**Figure 2:**
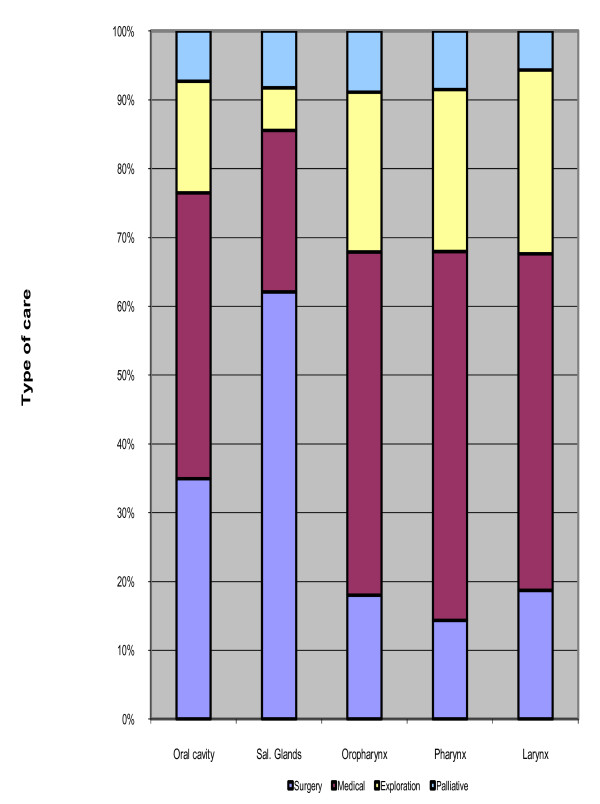
**Hospital stays distribution per category of care**.

In 2007, the management of head and neck cancers was responsible for 60 200 hospital stays and 287 846 chemotherapy or radiotherapy sessions. For stays, the most common type of management was medical care, which represented 48% of stays, especially for the management of patients with cancer of oropharynx, pharynx or larynx. 80% of these stays were performed in the public sector and the mean length of stay was 6 days. 6% of all medical stays were for chemotherapy. Surgery concerned 23% of stays and mainly concerned patients with cancer of oral cavity or oropharynx. 70% of these stays were seen in the public sector. Stays for exploration represented 22% of stays, were mainly performed in day-hospital setting and both concerned public and private sector. Palliative care concerned 8% of stays, 80% of them were performed in public sector and their mean length of stay was 16 days. The figure 2 presented the part of each type of management by cancer localization.

Among the 287 846 sessions extracted, 211 131 sessions (73%) were for radiotherapy. 83% of the sessions concerned men. The number of sessions by cancer localization is presented in table 1. Patients received an average of 18 sessions of chemotherapy or radiotherapy per year. Seventy-nine percent of radiotherapy sessions were made in public setting.

### Hospital cost

Table 2 gives the mean hospital cost per patient and the total annual cost per localization and per category of cost.

**Table 2:** Mean hospital cost per patient and total annual costs per localization and per category of cost (hospitalization, expensive drugs, outpatient and indirect costs) and from a payer perspective

	Mean hospital cost per patient (€)		Total annual cost (all patients) from payer perspective** (€)						
	**Public**	**Private**	**Hospital cost**	**Expensive drugs**	**Indirect cost**	**Outpatient cost**	Total**	**HPV positive cancer**	HPV related cost*

**Oral cavity**	7,673	3,054	71°558°167	7 727 519	18 395 395	33 013 172	130 694 253	23.5%	30 713 149

**Salivary glands**	6,151	2,929	9°728°585	991 317	2 063 391	4 488 257	17 271 550	-	-

**Oropharynx**	7,575	3,562	83°415°630	14 114 364	22 708 632	38 483 581	158 722 207	33 to 72%	52 378 328 to 114 279 989

**Pharynx**	7,580	3,468	66°449°315	10 907 202	17 570 038	30 656 216	125 582 771	-	-

**Larynx**	6,355	2,764	53°277°788	5 156 119	15 238 397	24 579 567	98 251 871	5 to 24%	4 912 594 to 23 645 249

**Total**	8,730	3,576	284°429°484	38 929 041	75 975 853	131 220 792	530 555 170	26%	137 944 344

* HPV related cost = prevalence × (hospital cost + expensive drugs + indirect cost + outpatient cost)

** Rehabilitation costs are not included in this estimate

The mean annual cost of hospitalization reached €7 842 per patient with head and neck cancers. However, a great heterogeneity was observed between public and private sector, cancer localization and type of management, as presented in table 2. For example, the mean annual cost per patient with H&N cancers varied from € 6 151 to € 7 673 in public hospitals, whereas it varied from €2 764 to €3 562 in the private sector. The annual cost of hospitalization was higher in men than in women, respectively equal at 8 038 € and 6 982 € on average. The type of management also influenced hospitalization costs: they were higher if the primary diagnosis was surgery or palliative care than for chemotherapy or radiotherapy. In 2007, total hospitalization cost for head and neck cancers in France amounted to €284 millions.

An expensive drug was administered in 53% of sessions for chemotherapy and 8% of stays for palliative care in 2008. The number of stays or sessions concerned by the administration of expensive drug varied according cancer localization, ranging from 46% for hypopharynx to 89% for lip. Most of these sessions or stays concerned men (85%). The global amount of expensive drug reached 38 929 041 €. Total hospital cost, including hospitalization and expensive drugs, then amounted to 323 millions € in 2007.

### Burden of HPV-related H&N cancers in 2007

Twenty-six percent of head and neck cancers are attributable to HPV infections [8], the overall HPV prevalence varying from 33% to 72% for oropharynx, 23.5% for oral cavity and to 5 to 24% for larynx (no published available data regarding the prevalence of HPV in salivary glands and pharynx other than oropharynx). Using these prevalence data, the annual number of patients hospitalized due to HPV-related H&N cancers was estimated to be 9 430. The annual burden of HPV-related cancer reached €138 million in 2007 (Table 2).

## Discussion

This survey was designed to estimate the annual number of patients hospitalized for head and neck cancers in France in 2007 and their related management costs. The study showed that the impact of head and neck cancers was important, as, in 2007 there were 36 268 patients hospitalised, corresponding to 60 200 hospital stays and 287 846 sessions of chemo- or radiotherapy. Oropharynx cancer was the most frequent (28% of patients), followed by oral cavity cancer (25% of patients). The study showed that annual hospital cost, (including hospitalisation and expensive drugs) of these patients appeared to be very high, close to 306 millions €. Men are particularly concerned by H&N cancers, as they represented 81% of all patients treated and 83% of all expenses (254 millions €).

Data were extracted from the PMSI database, as it presented a high exhaustiveness and a high level of quality. As discharge reports are the basis of hospitals funding and their reporting is mandatory, the PMSI database contained more than 98% of all public and private hospital stays in 2007, then limiting the sampling errors. Moreover, as all diagnoses are coded using the ICD-10 classification, all discharge reports are standardized, allowing to perform extraction of stays by disease. Coding errors is expected to be rare as this database constitutes the prospective financial system based on hospital activity, and since financial resources are directly related to the coded information. Coding quality in the PMSI is expected to be high and is guaranteed by controls performed annually in each hospital. The PMSI database is then widely used to provide relevant information on the burden of hospitalizations in France [24,25] and it can be used to evaluate the annual burden of hospitalization of head and neck cancers in France.

The PMSI database also presented few limitations. First, it was not possible to evaluate with precision the number of sessions of radiotherapy performed in the private setting, as they are not included in the PMSI database. This number was calculated using the SAE database, after the application of the ratio between public and private radiotherapy sessions. The calculation of the ratio was however not specific to head and neck cancers, but global for all cancers. Moreover, the global number of patients hospitalized for head and neck cancers in 2007 may be underestimated, as it was not possible to link the radiotherapy sessions performed in the private sectors with the stays from the PMSI database.

This administrative database only concerned patient who are hospitalized at least one time per year. Patients with head and neck cancers managed exclusively in an outpatient setting are not included in the present analysis, leading therefore to a potential underestimation of the total annual number of cases of H&N cancers. Moreover, despite presence of an identification number, it is not possible to distinguish incident cases (newly diagnosed patients, in active cancer treatment, with expensive cancer treatment cost) from the other cases (patients in maintenance treatment, with less expensive treatment cost). Thus, the estimated mean cost per hospitalized patient reflects the average cost of management of all cases admitted to hospital (prevalent cases). This may be lower than the cost per patient of management of new cases only, as shown by van Agthoven et al. (17), who demonstrated that the cost of H&N cancers was € 21 858 for the two years following diagnosis and it decreased to 423 € during the 10 years of follow-up. Our estimations are similar with those for European countries: the total cost to society from head and neck cancers ranged from € 5.5 millions in Greece (for 650 cases per year) to € 893 millions in Germany. It is however difficult to make a comparison, given the differences in terms of incidence and structure of the Sickness Fund.

The use of PMSI database leads to underestimation of the burden of disease, as it is restricted to hospitalization costs. Other categories of cost, like expensive drug, indirect or outpatient costs were estimated by using other sources of data. Cost of expensive and innovative drugs, like cetuximab or docetaxel, was estimated using the FICHCOMP database from the year 2008 (as this database was not yet available in 2007). It was also not possible to directly evaluate the indirect costs related to loss of productivity. In France, the valuation of indirect costs used the budgetary approach which relates to the budget impact for the National Sickness Fund. The monetary compensation served to patients to offset partially their loss of income. This amounted to €46.2 per day of sick leave (calculated by dividing the total amount of compensation paid in Year 2007 by the total number of sick leave days (data provided by the National Health Accounts)). In the present study, an estimation of indirect costs can be calculated using results from the INCa report 2007 [20], which showed that the proportion of patients who stop working because of head and neck cancers was 91% and the mean length of sick leave was 120 days. According to these assumptions, indirect costs amounted to about €76 millions. These indirect costs only represent the part supported by Sickness Fund. From a societal perspective, other indirect costs, due to premature death, would also have to be considered. Moreover, except for patients requiring a hospitalization, cost of management of side-effects of treatment for H&N cancers, like dysphagia or mucositis, which are usually managed in ambulatory care, were not evaluated in the present study. For example, the cost of treatment of dysphagia was evaluated at $153 to $710 [26], whereas it reached € 3 480 to 4 764 per episode of mucositis [27]. Moreover, Penel et al [28] estimated that 31% of patients presented nosocomial infections or pneumonia following H&N surgery, inducing an extra-cost of management estimated at respectively 16 000 and 17 000 €, as compared to patients without nosocomial infections or pneumonia. This extra-cost was not included in the present analysis, because of the risk of double-counting process (as many nosocomial infections are managed in hospital for H&N patients). Concerning the outpatient costs, as no specific data on outpatient management for head and neck cancers were available to our knowledge, we used estimates of the management costs for H&N tumours, published by Weill et al [29], reporting the frequency and cost of 30 long-term disorders in France based on an analysis of national health insurance reimbursement databases. We retrieved from this study that the outpatient costs (including visits, drugs, exams, transportation) represented 31.6% of global cost in patients suffering from head and neck cancers. In the present study, the annual outpatient cost can then be estimated at €131 million. The total cost of management of head and neck cancers, from a Payer perspective, then amounted to €531 million per year, including hospitalisation, expensive drugs, outpatient and indirect costs. Some costs, like rehabilitation cost, were not estimated as no data are available. For head and neck cancers patients who had devastating physical and functional changes, rehabilitation care plays an important role on restoring function and assisting the patient to achieve an acceptable quality of life. It then appeared important to evaluate their cost.

Given the considerable burden of disease induced by head and neck cancers, it appears important to identify strategies allowing to reduce costs and to save money. Nevertheless, at this time, there is no data indicating if prevention (reduction of alcohol and smoking, HPV incidence), screening, targeted treatment will allow to reduce the costs in the future. The cost-benefit of these strategies may be evaluated in the specific context of head and neck cancers.

There is a growing evidence for a causal role for HPV (mainly HPV 16) in a subset of H&N cancers [11]. The proportion of HPV-positive oral cancers has significantly increased over the past decades, in parallel with a decline in HPV-negative cancers [10,30]. HPV vaccination has demonstrated a high prophylactic efficacy against HPV 16 and 18 related pre-cancerous lesions of the cervix, vulvar and vaginal. One can expect that HPV vaccination might extend protection against other cancers associated to HPV 16 and 18. These study's data could provide useful information for decision-makers in order to assess the potential additional effect of the HPV vaccination on H&N cancers.

## Competing interests

This research was supported by an unrestricted research grant from Sanofi Pasteur MSD. The authors acknowledge medical writing support from Isabelle Borget, funded by Sanofi Pasteur MSD. Article-processing charge was also supported by Sanofi-Pasteur MSD.

## Authors' contributions

Each of the authors has participated in the preparation of this paper, has read and approved the final manuscript.
